# A Prenatally Ascertained *De Novo* Terminal Deletion of Chromosomal Bands 1q43q44 Associated with Multiple Congenital Abnormalities in a Female Fetus

**DOI:** 10.1155/2015/517678

**Published:** 2015-02-04

**Authors:** Carolina Sismani, Georgia Christopoulou, Angelos Alexandrou, Paola Evangelidou, Jacqueline Donoghue, Anastasia E. Konstantinidou, Voula Velissariou

**Affiliations:** ^1^Department of Cytogenetics and Genomics, The Cyprus Institute of Neurology and Genetics, 6 International Airport Avenue, Ayios Dometios, 2370 Nicosia, Cyprus; ^2^Department of Genetics and Molecular Biology, General, Maternity, and Pediatric Clinic Mitera, Erythrou Stavrou 6, 15123 Athens, Greece; ^3^Department of Pathology, Medical School, University of Athens, Mikras Assias 75, 11527 Athens, Greece

## Abstract

Terminal deletions in the long arm of chromosome 1 result in a postnatally recognizable disorder described as 1q43q44 deletion syndrome. The size of the deletions and the resulting phenotype varies among patients. However, some features are common among patients as the chromosomal regions included in the deletions. In the present case, ultrasonography at 22 weeks of gestation revealed choroid plexus cysts (CPCs) and a single umbilical artery (SUA) and therefore amniocentesis was performed. Chromosomal analysis revealed a possible terminal deletion in 1q and high resolution array CGH confirmed the terminal 1q43q44 deletion and estimated the size to be approximately 8 Mb. Following termination of pregnancy, performance of fetopsy allowed further clinical characterization. We report here a prenatal case with the smallest pure terminal 1q43q44 deletion, that has been molecularly and phenotypically characterized. In addition, to our knowledge this is the first prenatal case reported with 1q13q44 terminal deletion and Pierre-Robin sequence (PRS). Our findings combined with review data from the literature show the complexity of the genetic basis of the associated syndrome.

## 1. Introduction

Pure deletions of distal chromosome 1q result in a recognizable disorder described as 1q43q44 deletion syndrome (OMIM-612337, http://www.omim.org). Although clinical manifestations vary, most patients share characteristic features such as moderate-to-severe intellectual disability, limited to no speech, dysmorphic facial features including round face, prominent forehead, flat nasal bridge, hypertelorism, epicanthal folds, and low set ears. Hypotonia, poor growth, microcephaly, corpus callosum abnormalities (CCA), and seizures are also commonly present in these patients. The case we present in the current study is, to our knowledge, the first prenatal case with the smallest pure 1q43q44 deletion in a female fetus molecularly and phenotypically characterized and the first reported case with an association with PRS. The detailed autopsy and genetic analysis results allow further characterization, clinical correlation, and/or genotype-phenotype correlation, as well as comparison with previously described prenatal and postnatal cases.

## 2. Case Report

**Figure 1:**
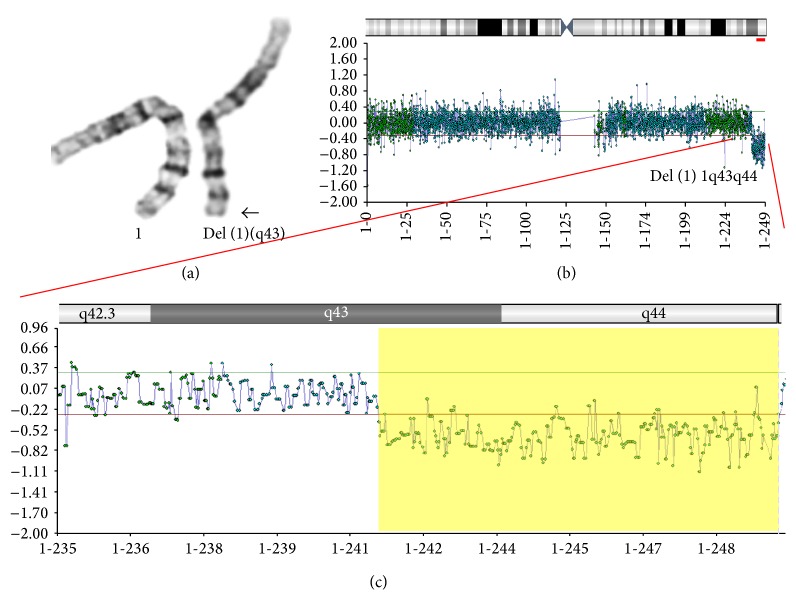
(a) Prenatal fetal karyotype. The prenatal fetal karyotype revealed a 1q43 deletion most probably terminal. Arrow points to the deleted region of the long arm of chromosome 1. (b) Array-CGH results indicating the terminal deletion chromosome 1. (c) Array-CGH analysis illustrating in depth the* de novo* terminal deletion (highlighted) of approximately 8 Mb in size on the long arm of chromosome 1 at chromosomal band 1q43 extending to band 1q44 (location: 241,178,091–249,224,121 using build GRCh37 (hg19)).

A 32-year-old pregnant woman was referred to our lab at 22 weeks of gestation for chromosomal investigation by karyotype analysis after amniocentesis, requested due to abnormal ultrasound findings. The prospective parents are both of Greek origin and apparently healthy. This was their first pregnancy (gravida 1, para 0) and no previous medical or obstetrical history was recorded. The pregnancy had been conceived spontaneously and was unremarkable until this point. Routine ultrasound examination at 22 weeks of gestation revealed choroid plexus cysts (CPCs) and a single umbilical artery (SUA). Chromosomal analysis was performed on amniotic fluid cells. Conventional GTG-banding at 550 band level was applied and revealed a female fetus with a borderline visible deletion on distal 1q, most likely terminal (46,XX,del(1)(q43), Figure 1(a)). Parental karyotypes revealed that the deletion was* de novo*. MLPA was subsequently performed using the P036 and P069 subtelomeric probe mixes (MRC-Holland) on DNA isolated from cultured amniotic fluid cells and confirmed the telomeric nature of the 1q deletion (data not shown). To further delineate the breakpoint of the deletion array-CGH (Comparative Genomic Hybridization) was carried out using the Cytochip Oligo array (BlueGnome-version 1.1) with 105,000 oligos according to the recommendations of the manufacturer. Array-CGH analysis confirmed the results, revealing a female profile with a deletion of approximately 8 Mb in size on the long arm of chromosome 1 from chromosomal band 1q43 extending to 1q44 (location: 241,178,091–249,224,121 using build GRCh37 (hg19), Figures 1(b) and 1(c)). No other copy number changes were detected by array-CGH indicating a pure deletion of the region. The deleted region contains 23 OMIM genes listed in Table 1.

Genetic counseling was offered to the couple and termination of the pregnancy was decided at 28 weeks of gestation. The female fetus was sent for autopsy (a written consent was also obtained from the couple for the publication including fetopsy photos).

**Figure 2:**
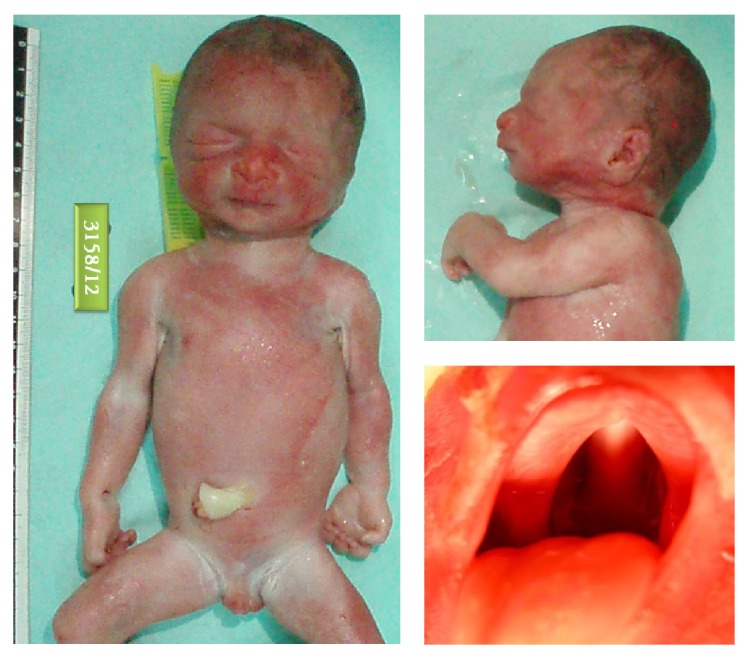
Autopsy findings revealed multiple congenital malformations. The figure shows microretrognathia and U-shaped cleft palate (Pierre-Robin sequence) as well as a large clitoris with normally formed labia.

At autopsy the fetus was found to be symmetrically growth restricted, weighing 838 g (below the 10th centile for 28 weeks-gestation) and measuring 33.5 cm in crown-heel length, 22.5 cm in crown-rump length, and 46.5 cm in foot length, the body measurements being more appropriate for 25-week gestation. The head circumference (22.8 cm) fell below the 1st centile for 28-week gestation. External craniofacial features included microcephaly and microretrognathia with U-shaped clefting of the hard and soft palate (Pierre-Robin sequence) (Figure 2). The tip of the tongue was noted to be mildly bifid. The clitoris appeared large, with normally formed and sized labia while the anogenital distance appeared shortened. Internally, dissection of the heart revealed an atrial septal defect (ASD) secundum type. The brain weight was low (84 g; ref. for 28/40 weeks 147 g) corresponding to 23-week gestation, and the brain-to-liver-weight ratio was reduced (1.69; ref. 2.5–4), altogether indicating micrencephaly. The cerebellar vermis appeared small. Choroid plexus cysts seen at prenatal ultrasound at 22-week gestation were not confirmed, likely to have resolved in the meantime. Microscopy showed neuroglial migration defects in the periventricular and subcortical cerebral white matter. The placenta weighed 169 g (around the 25th centile for 28/40 weeks) and the fetoplacental ratio was within normal range. Histology showed abnormal development of the placental parenchyma, with uneven villous maturation and features of fetal obstructive vasculopathy. The umbilical cord had 3 vessels, but one umbilical artery was seen to be collapsed, showing luminal occlusion and no evidence of blood flow at the fetal edge.

## 3. Discussion

The prenatal case presented in this study involves a terminal 1q43q44 deletion which in postnatal cases has been associated with a syndrome with specific clinical features (OMIM-612337, http://www.omim.org). Although manifestations may vary, our fetus had features overlapping the 1q43q44 deletion phenotype, as expected. Such characteristics are microcephaly, microretrognathia, cleft palate, cardiac defect, and small cerebellar vermis. Only three cases of terminal 1q deletions have been described during prenatal diagnosis. The first was detected in a fetus at 19 weeks with omphalocele, cerebral ventriculomegaly, and increased nuchal fold with a breakpoint at 1q41 [1]. The second was detected in a fetus at 21 weeks with hydrocephalus, ventriculomegaly, and corpus callosum agenesis with a breakpoint at 1q42.3 [2]. The third, with a breakpoint at 1q43, was detected by chorionic villous sampling (CVS) in a 12-week fetus with severe microgenia, nasal bone aplasia, SUA, cardiac anomaly, and hyperechogenic bowel. At 16 weeks, the suspected structural abnormalities were confirmed and in addition intrauterine growth retardation (IUGR) and microcephaly were observed [3]. In the first two cases the deletions were considerably larger than in our case and easily detected during cytogenetic analysis. In the third case, although there are common clinical findings with our case such as microcephaly, SUA, and IUGR, autopsy was not performed and a detailed comparison of the phenotype between the two cases is not possible. For example, the authors report severe microgenia, which at autopsy could prove to be microretrognathia with clefting of the hard and soft palate (PRS), also present in our case. Furthermore, the breakpoint of the deletion was only determined by chromosomal analysis and it appears that the deletion is again significantly larger than ours. Finally, array CGH studies were performed only in the second case where the size of the deletion was estimated at 13.4 Mb, which is significantly larger than that in our case (8 Mb). The antenatal features of the previously three reported del(1q) syndrome prenatal cases and of our present case are summarized in Table 2.

Two recent studies [5, 6] in patients with 1q43q44 microdeletion clarified the phenotype/genotype correlation and proposed three distinct critical regions. The first encompassing* ZNF238* was associated with corpus callosum anomalies (CCA) and the second includes* AKT3* with microcephaly (MIC), while the third contains the two coding genes* FAM36A, HNRNPU* and the noncoding gene* NCRNA00201* with seizures. The implication of* ZNF238* in CCA has also been supported by the study of a patient with 1q44 microdeletion and dysmorphic features, seizures, hypotonia, marked developmental delay, and dysgenesis of the corpus callosum [7]. In the case presented here,* AKT3*,* ZNF238*,* FAM36A*, and* HNRNPU* are all included in the deletion. Fetoscopy showed evidence of microcephaly which is in agreement with the implication of* AKT3* in MIC as has already been proposed by others [5, 6, 8]. However, although* ZNF238 *is absent, CCA was not observed, demonstrating the complexity of CCA genetics. Incomplete penetrance associated with deletion of* ZNF238* could be an explanation. This has also been proposed in a study in which two patients with moderate-to-severe intellectual disability, craniofacial anomalies, and seizures carrying 1q44 microdeletion including both* ZNF238* and* AKT3* both had MIC, but only the one presented with CCA [8].

The size of the deletion in our case is comparable to that of a boy with intellectual disability and multiple anomalies where the 1qter deletion was not microscopically visible [4]. At 20-week gestational age a cystic structure in the gastric region was seen on ultrasound which subsequently disappeared, while from 30 weeks IUGR was present. After birth major anomalies were detected and at the age of 5 years severe intellectual disability was also observed. Common features with our case are IUGR, microretrognathia, microcephaly, hypoplastic vermis, and genital abnormalities. However, the mapping of the deletion was performed by microsatellite marker analysis and not array CGH and consequently the exact comparison cannot be made, although the two cases appear to be very similar (Table 2).

The postmortem examination in our case also revealed the presence of PRS, that is, the combination of microretrognathia and posterior soft palate cleft. To date this has been strongly associated with* SOX9 *either directly or by position effect [9, 10]. It has also been associated with specific gene mutations and various chromosomal abnormalities including deletions, translocations, and duplications [11]. Recently, it has been associated with microdeletion 4q21, microdeletion 5q23, and microduplication 16p13.3 [12–14]. To our knowledge, this is the first reported case with pure 1q43q44 deletion and PRS. There is a single report on a family with cerebellar hypoplasia and PRS, partially resembling our case clinically; however array CGH and subtelomeric FISH revealed no chromosomal imbalances [9]. Our case further supports the fact that PRS conceals considerable etiological heterogeneity and that various chromosomal regions harbor genes responsible for the PRS phenotype, including 1q43q44.

In addition to PRS, the present case is the first in which neuroglial migration defects were detected in the brain, whereas in previous deletion 1q43q44 cases brain abnormalities included delayed myelination, cerebral atrophy, and hydrocephalus [2, 8].

Our findings are in agreement with most reports, the main disagreement with previous reports relates to gene* ZNF238* which although is included in the deleted region no corpus callosum abnormalities were identified. The possibility of identifying specific ultrasonographic markers for 1q43q44 deletion during pregnancy is not clear at the moment because of the limited number of cases described prenatally.

All the above findings highlight the complexity of gene implication/interaction, reflected in the difficulty of genotype-phenotype correlations, and the fact that probable additional mechanisms such as incomplete penetrance, variable expressivity, or multigenic factors may also influence phenotypic expression. Reporting such cases may contribute to better understanding of these issues.

## Figures and Tables

**Table 1:** Genes included in the deleted region and associated phenotypes.

Gene/locus name	Gene/locus MIM number	Phenotype/gene function
*RGS7 *	602517	Inhibits signal transduction by increasing the GTPase activity of G protein alpha subunits
*FH, HLRCC, MCUL1 *	136850	Leiomyomatosis and renal cell cancer. Fumarase deficiency
*KMO *	603538	Catalyzes the hydroxylation of L-kynurenine (L-Kyn) to form 3-hydroxy-L-kynurenine for synthesis of quinolinic acid
*OPN3, ECPN *	606695	Opsins are members of the guanine nucleotide-binding protein (G protein)-coupled receptors that are expressed in extraocular tissues
*CHML, REP2 *	118825	Binds unprenylated Rab proteins
*EXO1, HEX1 *	606063	5′->3′ double-stranded DNA exonuclease activity
*CEP170, KIAA0470 *	613023	Plays a role in microtubule organization
*SDCCAG8, CCCAP, SLSN7 *	613524	Senior-Loken syndrome 7
*AKT3, PKBG, MPPH *	611223	Megalencephaly-polymicrogyria-polydactyly-hydrocephalus syndrome
*ZBTB18, ZNF238, RP58, MRD22 *	608433	Mental retardation, autosomal dominant 22
*ADSS *	103060	Has an important role in the *de novo* and salvage pathway of purine nucleotide biosynthesis
*DESI2, PPPDE1 *	614638	Protease which may deconjugate SUMO from some substrate proteins
*COX20, FAM36A *	614698	Protect as-yet-unassembled Cox2 from degradation
*HNRNPU *	602869	Component of the CRD-mediated complex that promotes MYC mRNA stabilization. Binds to pre-mRNA.
*KIF26B *	614026	Essential for embryonic kidney development
*SMYD3 *	608783	Histone methyltransferase
*TFB2M *	607055	Required for basal transcription of mitochondrial DNA, probably via its interaction with POLRMT and TFAM
*CNST *	613439	Required for targeting of connexins to the plasma membrane
*AHCTF1, ELYS *	610853	Required for the assembly of a functional nuclear pore complex on the surface of chromosomes as nuclei form at the end of mitosis
*ZNF124 *	194631	Affiliated with the lncRNA class and may be involved in transcriptional regulation
*ZNF496, NIZP1, ZFP496 *	613911	DNA-binding transcription factor that can both act as an activator and a repressor
*NLRP3, CIAS1, FCU, FCAS, NALP3, PYPAF1 *	606416	CINCA syndrome, Cold-induced autoinflammatory syndrome, familial Muckle-Wells syndrome
*OR13G1 *	611677	Odorant receptor (potential)

**Table 2:** Clinical features and genetic findings in one postnatal with a very similar deletion size and three prenatal reported cases with deletions spanning chromosomal region 1q41 to 1q44 and comparison with our case.

	Rotmensch et al. [1] prenatal	Chen et al. [2] prenatal	Wagner et al. [3] prenatal	van Bever et al. [4] postnatal	Our case prenatal
Karyotype	46,XY, del(1)(q41)	46,XX, del(1)(q42.3)	46, XX, del(1)(q43)	46, XY	46, XX, del(1)(q43q44)
Light microscope	Detected	Detected	Detected	Not detected	Detected
Array CGH	Not performed	13.4 Mb	Not performed	7.7–8.1 Mb^§^	8 Mb
Parental karyotypes	Normal	Normal	Normal	Normal	Normal
Gestational age	2nd trimester	2nd and 3rd trimester	1st and 2nd trimester	2nd and 3rd trimester	2nd and 3rd trimester
IUGR	—	+	+	+	+
Increased nuchal translucency/fold	+	—	—	—	—
Nasal bone absence/hypoplasia	—	—	+	+	—
Hyperechogenic bowel	—	—	+	—	—
Single umbilical artery	—	—	+	—	+
Omphalocele	+	—	—	—	—
Microcephaly/micrencephaly	∗	—	+	∗	∗
Micrognathia/microgenia	—	—	+	∗	∗
Microretrognathia	—	—	—	∗	∗
Cleft palate	—	—	—	—	∗
Hydrocephalus	—	+	—	—	—
Cerebral anomalies	+	—	—	∗	∗
Corpus callosum agenesis/hypoplasia	—	+	—	∗	—
Hypoplastic vermis	—	—	—	∗	∗
Choroid plexus cysts	—	—	—	—	+
Urogenital anomalies	—	—	—	∗	∗
Cardiac anomaly	—	+	+	∗	∗
Fetopsy	—	—	—	∗∗	+

^§^Performed by microsatellite marker analysis; IUGR: intrauterine growth retardation.

—: not specifically mentioned or undetected; ∗: initially detected at birth or after termination of pregnancy by observation only or fetopsy; ∗∗: born; +: present.
